# Anomalous negative longitudinal magnetoresistance and violation of Ohm's law deep in the topological insulating regime in Bi$_{1-x}$Sb$_x$

**DOI:** 10.1038/s41598-021-87780-0

**Published:** 2021-04-22

**Authors:** Amit Vashist, R. K. Gopal, Yogesh Singh

**Affiliations:** Department of Physical Sciences, Indian Institute of Science Education and Research (IISER) Mohali, Knowledge City, Sector 81, Mohali, 140306 India

**Keywords:** Physics, Condensed-matter physics

## Abstract

Bi$_{1-x}$Sb$_x$ is a topological insulator (TI) for $x \approx 0.03$–0.20. Close to the Topological phase transition at $x = 0.03$, a magnetic field induced Weyl semi-metal (WSM) state is stabilized due to the splitting of the Dirac cone into two Weyl cones of opposite chirality. A signature of the Weyl state is the observation of a Chiral anomaly [negative longitudinal magnetoresistance (LMR)] and a violation of the Ohm’s law (non-linear $I{-}V$). We report the unexpected discovery of Chiral anomaly-like features in the whole range ($x = 0.032, 0.072, 0.16$) of the TI state. This points to a field induced WSM state in an extended *x* range and not just near the topological transition at $x = 0.03$. Surprisingly, the strongest Weyl phase is found at $x = 0.16$ with a non-saturating negative LMR much larger than observed for $x = 0.03$. The negative LMR vanishes rapidly with increasing angle between *B* and *I*. Additionally, non-linear *I*–*V* is found for $x = 0.16$ indicating a violation of Ohm’s law. This unexpected observation of a strong Weyl state in the whole TI regime in Bi$_{1-x}$Sb$_x$ points to a gap in our understanding of the detailed crystal and electronic structure evolution in this alloy system.

Condensed matter physics has taken an exciting turn after the discovery of two and three dimensional (2D/3D) topological materials possessing a nontrivial bulk band structure characterized by topological invariants^1–4^. In particular, Topological (Dirac or Weyl) semi-metals (DSM, WSM), which possess bulk bands with linear electronic dispersions in all three momentum directions, have attracted immense recent interest^5–14^. In DSMs, doubly degenerate Dirac cones (of opposite chirality) exist which are protected by time reversal (TRS) and inversion (IS) symmetry. Na$_3$Bi^9^ and Cd$_3$As$_2$^14^ are examples of the most studied DSM materials. Breaking of any of the above symmetries leads to a splitting of the Dirac cones into a pair of Weyl cones of opposite chirality, turning the DSM into a WSM. A WSM state has been shown for example, in the mono-arsenides TaAs and NbAs where the IS is broken. A TRS broken WSM state has been found for example in YbMnBi$_2$^15^. Another route to designing a WSM has been demonstrated for the half-Heuslar material GdPtBi which, in zero magnetic fields, is a gapless semiconductor with quadratic bands. On application of a magnetic field, the quadratic bands are Zeeman split and Weyl nodes are formed at the crossings of the Zeeman split bands^16^.

WSMs show several anomalous and potentially technologically useful transport properties such as very large charge carrier mobility $\mu$ (and hence a large electronic mean free path $l_e$), low carrier density, giant linear magnetoresistance (MR), anomalous Hall effect, and strong anisotropy in the MR with a negative longitudinal MR (LMR) when the magnetic field *B* is applied parallel to the current *I*^7, 17–22^. The negative LMR is a consequence of the Adler–Bell–Jackiw Chiral anomaly^23,24^ predicted for Weyl Fermions in parallel magnetic and electric fields *B*||*E*^25^. A crucial signature of the Chiral anomaly is that the negative LMR is extremely sensitive to the angle between *B* and *E* and is rapidly suppressed as the angle between *B* and *E* is increased. The Chiral anomaly has been observed in the WSMs TaAs^26^, Na$_3$Bi^27^, Cd$_3$As$_2$^28,29^, and GdPtBi^16^ among others.

The alloy system Bi$_{1-x}$Sb$_x$ has also been a very fruitful playground for the observation of various Topological states. In fact the first 3D Topological Insulator state was experimentally verified in Bi$_{1-x}$Sb$_x$ (x ~0.03-0.04). Bi is a topologically trivial semi-metal. As Sb is introduced, a band inversion occurs at $x \approx 0.03$ signalling a Topological phase transition into a TI state beyond $x \approx 0.03$. The TI phase spans a large range $x = 0.03$–0.22 in the Bi$_{1-x}$Sb$_x$ system^30–35^.

At the Topological transition point $x = 0.03$, the material is a DSM which changes to a WSM state on the application of a magnetic field. Indeed the Chiral anomaly in the *B*||*E* configuration with a strongly angle dependent negative LMR has been observed for the $x = 0.03$ material^36^. Additionally, a violation of the Ohm’s law has been observed for the $x = 0.05$ material which also lies close to the Topological phase transition into a TI state, and has been argued to be a consequence of the Chiral anomaly^37^. This study also concluded that no negative LMR was observed for samples far away ($x > 0.05$) from the Topological transition^37^. Thus, Bi$_{1-x}$Sb$_x$ alloys host a TI state in an extended range $x = 0.03$–0.22, a DSM state at $x = 0.03$, and a WSM state for $x = 0.03$ when TRS is broken by the application of a magnetic field. Very recently, the Sb rich side of Bi$_{1-x}$Sb$_x$ has been investigated theoretically and two new WSM phases have been predicted at large Sb doping of $x = 0.5$ and $x = 0.83$ due to the formation of IS breaking structures^38–40^. This suggests that new surprises are yet to be discovered in this well studied system as shown in previous preliminary magneto-transport results^35^.

In this article we have made a detailed magneto-transport study on six high quality single crystals of Bi$_{1-x}$Sb$_x$, covering the whole TI range $x = 0.032$–0.16. We provide clear evidence that in addition to the crystal at $x = 0.032$ which is at the Topological phase transition, samples at $x = 0.072$ and 0.16 also show Chiral anomaly like signatures with a negative LMR which is strongly suppressed on increasing the angle between *B* and *I*. Additionally, non-linear *I*–*V* is observed for $x = 0.16$ crystals only when *B*||*I*. The negative LMR for the $x = 0.072$ sample is weaker than that for $x = 0.032$ and $x = 0.16$ samples. The negative LMR for $x = 0.16$ is the strongest. These observations strongly indicate that a WSM state exists for other compositions *x* even far away from the Topological phase transition at $x = 0.03$. These are unexpected results given that beyond $x \approx 0.04$, the Bi$_{1-x}$Sb$_x$ system is considered to be a TI with no bulk bands. Our discovery calls for a careful relook at existing ARPES and STM data to identify the exact locations of the discovered Weyl nodes. At the end we discuss various mechanisms which could lead to the stabilization of the WSM state seen by us.

## Experimental

Single crystals of Bi$_{1-x}$Sb$_x$ ($0\le x \le .16$) were grown using a modified Bridgeman technique^35^. Stoichiometric amounts of Bi (5N) and Sb (5N) shots were sealed in a quartz tube under vacuum. The quartz tube was placed vertically in a box furnace and heated to $650~^o$C in 15 hrs, kept there for 8 hrs, and then slowly cooled to $270~^o$C over a period of five days for crystal growth. The crystals are kept at $270~^o$C for seven days for annealing and homogenization. Large shiny crystals could be cleaved from the resulting boul. The crystals grow with the *c*-axis along the axis of the cylindrical quartz tube. The structure of the crystals was confirmed using powder x-ray diffraction (PXRD) on crushed crystals which was collected at room temperature using a Rikagu diffractometer (Cu K$\alpha$). Chemical analysis of the crystals was done using energy dispersive spectroscopy (EDS) on a JEOL scanning electron microscope (SEM). Details of the crystal homogeneity estimated from EDS measurements is given in the supplementary materials. Electrical transport was measured on rectangular crystalline pieces of length 3 mm, width 0.5 mm and thickness $\approx 100~\mu$m. AC measurement technique with a current of amplitude $I = 2$ mA was used for all electrical measurements. The contacts on the single crystals were made using high-quality silver paint in a collinear geometry. The angle dependent magnetoresistance measurements were made using the horizontal rotator option of a Quantum Design physical property measurement system (PPMS).

## Results

**Figure 1:**
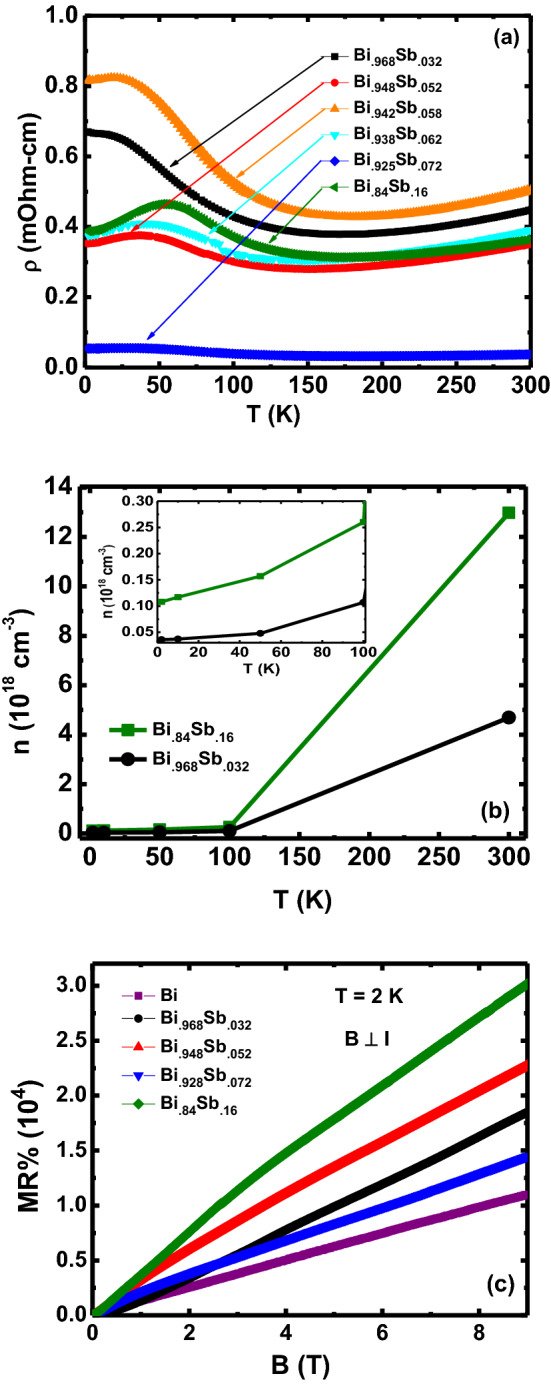
(Color online) (**a**) Resistivity $\rho$ vs temperature *T* for Bi$_{1-x}$Sb$_x$. (**b**) The carrier density *n* versus *T* for $x = 0.032, 0.16$ samples. Inset shows n(T) for T < 100 K. (**c**) Transverse magneto resistance *MR* vs *B* at $T = 2$ K for Bi$_{1-x}$Sb$_x$. The *MR* for all *x* is positive and linear at high *B*.

We first show that our Bi$_{1-x}$Sb$_x$ ($0.032\le x \le .16$) crystals show the expected transport properties reported previously. Figure 1a shows the temperature dependent electrical resistivity $\rho (T)$ data for Bi$_{1-x}$Sb$_x$ with $x \ge 0.032$. For all these materials the qualitative *T* dependence is similar. We observe small band-gap semi-conducting behaviour at high temperatures $T > 50$ K, while the resistivity saturates to a nearly constant value at low temperatures. This behaviour is consistent with previous reports on Bi$_{1-x}$Sb$_x$ ($0.028\le x \le 0.20$) crystals^41^. The residual resistivity value in a gapped system like Bi$_{1-x}$Sb$_x$ has been attributed to a small amount of impurities acting as shallow donors which make the materials slightly *n*-doped^41^. In principal, one does not expect a monotonic change in the resistivity for the Sb substituted samples. The substitution of Sb for Bi is not expected to lead to any charge doping, since they have the same outer shell electron count. The transport can depend on a combination of carrier concentration and mobility and their temperature dependences. Additionally, the observed trend, or lack of it, suggests a combination of chemical pressure and disorder effects. Figure 1b shows the carrier density *n* estimated from Hall measurements, as a function of *T* for the $x = 0.032, 0.16$ samples. The *n* drops by about two orders of magnitude on cooling from 300 K to 2 K indicating the gapped nature of the crystals. An Arrhenius fit ($n \propto exp^{-\Delta /k_BT}$) to the data gives a band-gap of $\approx 18$ and $\approx 30$ meV for the $x = 0.032$ and 0.16 samples, respectively. These values of the band-gap are consistent with expectations from previous reports^30,41^.

Large conventional transverse magnetoresistance with magnetic field perpendicular to the current direction has been reported in the TI state. Transverse magnetoresistance percent (MR%) for Bi$_{1-x}$Sb$_x$ with $B \perp I$ is shown in Fig. 1c. We observe large non-saturating positive MR for all samples which is linear up to the largest *B*, consistent with previous observations on samples close to $x = 0.04$. Thus our samples show behaviour consistent with them being in the TI state. We note that the MR% is largest for the $x = 0.16$ sample and is much larger than reported previously for the $x = 0.04$ samples close to the trivial to Topological insulator transition.

**Figure 2:**
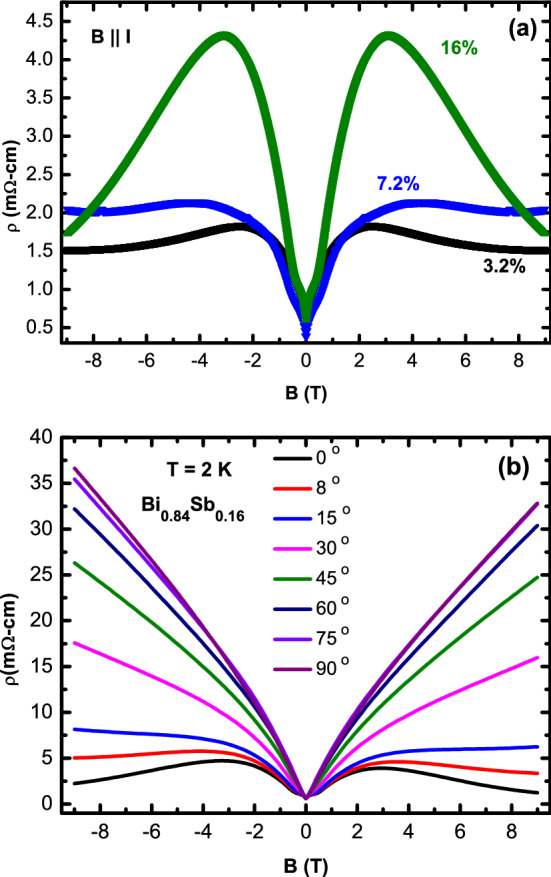
(Color online) (**a**) Resistivity $\rho$ vs magnetic field *B* at $T = 2$ K with *B*||*I* for Bi$_{1-x}$Sb$_x$ ($x = 3.2, 7.2, 16$ %). (**b**) $\rho$ vs *B* at $T = 2$ K for the $x = 16$% sample with *B* applied at various angles to the current *I*. The negative LMR seen clearly for *B*||*I* is rapidly suppressed as the angle between *B* and *I* increases.

We now show evidence for the Chiral anomaly in the whole TI region. Figure 2a shows the resistivity $\rho$ vs magnetic field *B* measured at temperature $T = 2$ K with the magnetic field *B* applied parallel to the electrical current *I* for Bi$_{1-x}$Sb$_x$ ($x = 0.032, 0.072, 0.16$). These three samples are located (i) close to the transition from the normal insulator to a TI ($x = 0.032$), (ii) in the middle of the *x* range in which a TI has been shown to exist ($x = 0.072$), and (iii) near the end of the range of the TI state where the semi-conducting band-gap is largest ($x = 0.16$). For the $x = 0.032$ sample, we observe that after the initial increase in $\rho$ at small *B* most likely arising from weak anti-localization (WAL), the $\rho$ turns down and starts decreasing upto the highest *B* measured. This is the negative longitudinal (*B*||*I*) magnetoresistance (NLMR) or the Chiral anomaly. For $x = 0.032$ the Bi$_{1-x}$Sb$_x$ is situated close to the Dirac semi-metal state and the application of a magnetic field leads to time-reversal symmetry breaking and hence the Dirac cone is expected to split into a pair of Weyl nodes and so the Chiral anomaly (NLMR) for this composition is expected and has been observed previously as well for Bi$_{0.096}$Sb$_{0.04}$^36^ and Bi$_{0.095}$Sb$_{0.05}$^37^ samples.

What is surprising is that we observe Chiral anomaly-like signatures even for samples far away from $x = 0.032$. Figure 2a also shows the $\rho$ vs *B* data for $x = 0.072$ and $x = 0.16$. The $x = 0.072$ sample shows a negative LMR above $B \sim 4$ T suggesting a Weyl state for this *x* too. However, the $\rho$ data for $x = 0.16$ shows the strongest negative LMR compared to even the $x = 0.032$ sample. The NLMR keeps increasing up to the highest magnetic fields measured $B = 9$ T. This strongly suggests that a WSM state exists for $x = 0.16$ as well. The NLMR for $x = 0.16$ is completely novel and has not been observed before.

Extrinsic mechanisms like current jetting can also lead to a NLMR as has been demonstrated previously^29^. To rule out such effects, we check for a crucial signature of the Chiral anomaly which is the strong dependence of the NLMR on the angle between *B* and *I*. Figure 2b shows the angle dependence of the NLMR for the $x = 0.16$ sample. We observe that for angle $= 0$ (*B*||*I*), the NLMR is the largest and as the angle between *B* and *I* is increased, the magnetoresistance quickly increases and changes to completely positive MR for angle $\ge 8~^o$. This strong sensitivity of the NLMR to the angle between *B* and *I* is strong evidence of the Chiral anomaly for the $x = 0.16$ sample and points to it being in the WSM state.

**Figure 3:**
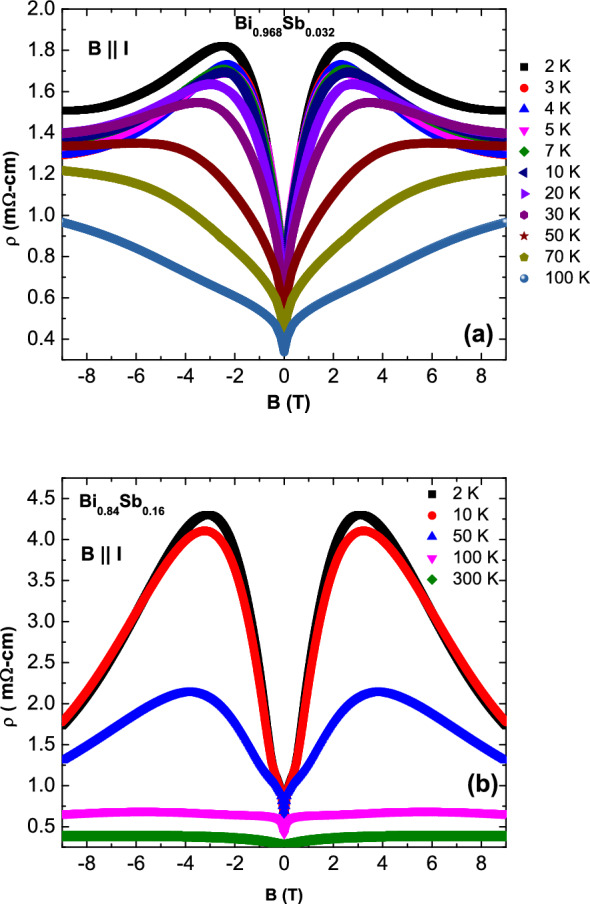
(Color online) Temperature dependence of the negative LMR for the (**a**) $x = 0.032$ and (**b**) $x = 0.16$ samples.

We provide further evidence against geometrical mechanisms like current-jetting by tracking the temperature dependence of the NLMR. The geometrical-effect-driven NLMR is expected to become strongest at intermediate temperatures where a matching between the temperature dependent mean free path and the separation between voltage contacts occurs. A Chiral anomaly driven NLMR on the other hand becomes stronger as temperature is reduced^30, 37^. The *T* dependence of the NLMR for the $x = 0.032$ and 0.16 samples as shown in Fig. 3a,b, respectively. The NLMR increases strongly and monotonically as one goes to lower temperatures strongly indicating the intrinsic nature of the NLMR for both samples.

**Figure 4:**
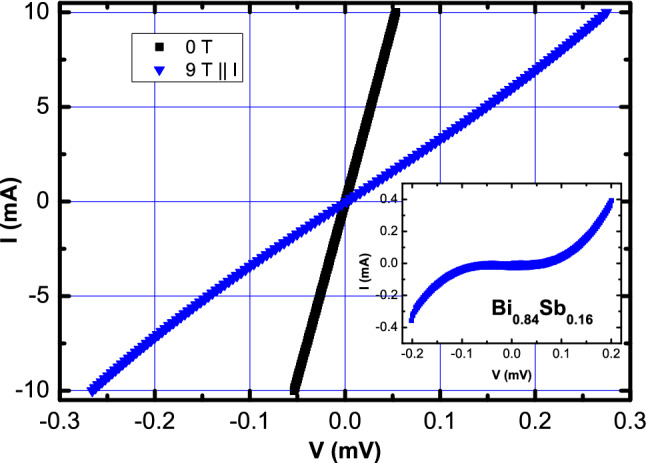
(Color online) *I*–*V* curve for the $x = 0.16$ sample measured at $T = 2$ K in $B = 0$ and in $B = 9$ T for *B*||*I*.

Further confirmation of anomalous transport behaviour expected for a WSM is obtained for the $x = 0.16$ sample from the observation of a violation of the Ohm’s law. Figure 4 shows the *I*–*V* curve for the $x = 0.16$ sample measured at $T = 2$ K in $B = 0$ and in $B = 9$ T for *B*||*I*. The $B = 0$ data are completely linear as expected in conventional metals. The *I*–*V* data at $B = 9$ T with $B \perp I$ were also measured (not shown) and were found to be completely linear. For *B*||*I*, the LMR configuration, a clear non-linearity can be seen in the *I*–*V* curves. To highlight the non-linearity, we have subtracted the linear part of the *I*–*V* curve obtained by a fit to the $I \le 2$ mA data. The resulting *I*–*V* curve obtained at 9 T is shown in Fig. 4 inset and clearly shows a non-linear behaviour. This clear violation of the Ohm’s law has previously been reported only for the $x = 0.05$ samples close to the Topological transition boundary^37^.

## Summary and discussion

We have studied the magneto-transport properties of Bi$_{1-x}$Sb$_x$ single crystals for $x = 0.032$–0.16 spanning almost the whole range of Sb substitution for which Bi$_{1-x}$Sb$_x$ has previously been shown to be a Topological Insulator (TI). The transition from trivial insulator to a TI occurs at $x \approx 0.03$. Samples at this topological phase transition are Dirac semi-metals (DSM) and the application of a magnetic field breaks time reversal symmetry and hence splits the Dirac cone into two opposite chirality Weyl cones displaced in momentum space along the magnetic field direction turning the material into a Weyl semi-metal (WSM). A smoking gun signature of a WSM state is the observation of a Chiral anomaly i.e. a negative longitudinal magnetoresistance (LMR) when a magnetic field *B* is applied along the current *I* direction. This negative LMR is expected to be strongly suppressed as the angle between *B* and *I* is increased. Additionally, a non-linear *I*–*V* has been reported for the WSM state at $x = 0.05$. We indeed observe the Chiral anomaly for our $x = 0.03$ crystals in agreement with previous reports on samples with $x = 0.04$ and 0.05. Unexpectedly, we also find strong evidence of a WSM state for the $x = 0.16$ sample, which is close to the end of the TI state. In particular, we find a negative LMR even stronger than the $x = 0.032$ crystal. The NLMR for the $x = 0.16$ sample is strongly suppressed with increasing angle between *B* and *I*. Further, the magnitude of the NLMR increases monotonically as the temperature is lowered. The angle and temperature dependence of the NLMR strongly rule out extrinsic mechanisms like current-jetting. Additionally for the $x = 0.16$ crystal, we observe a non-linear *I*–*V* for *B*||*I* but not for $B \perp I$. A weaker but clearly observable negative LMR is also found for the $x = 0.072$ sample. These observations strongly indicate that in addition to $x = 0.03$, a WSM state exists for *x* values expected to be deep in the TI state.

How can these mutually exclusive states, TI and WSM exist in the same material. There have been recent DFT studies of the structure and atomic arrangements of Bi and Sb in Bi$_{1-x}$Sb$_x$^38–40^. It has been shown that as Sb concentration increases, an inversion symmetry (IS) breaking structure becomes near degenerate to the IS preserving structure and can be stabilised. Globally the two structures are the same and they differ only in the atomic arrangement of Bi and Sb^38–40^. It was further shown that these IS breaking structures host WSM phases^38–40^ which can be tuned by pressure^38,39^. In addition to the WSM phases predicted in the IS breaking structures, a recent magnetic field dependent band-structure study has discovered a field induced WSM state induced by Zeeman splitting for the $x \approx 11, 15\%$ materials. This is experimentally supported by the observation of a thermal Chiral anomaly^42^. The exact mechanism of stabilising the WSM state in our materials are still unknown. However, these recent studies^38–40,42^ suggest that either an IS breaking with increasing *x* or a closing of the small ($\sim 100$ meV) indirect band-gap by a magnetic field, or a combination of both, could lead to the WSM states whose evidence we see in the NLMR and non-linear *I*–*V*. Since the global crystal structure of our Bi$_{1-x}$Sb$_x$ crystals is the same as Bi, methods like the pair distribution function analysis of x-ray data could help shed light on the atomic distribution of Bi and Sb to look for IS breaking structures. Additionally, TEM may also be useful to look at the local atomic arrangement as well. Finally, evolution of the electronic structure with magnetic field is desirable to elucidate the origin of the observed WSM states.

## Note added

After submission, we became aware of a report^42^ on observation of the Chiral anomaly in thermal transport measurements in the field induced Weyl state of Bi$_{1-x}$Sb$_x$ single crystals for $x = 0.11$ and 0.15. These results are consistent with our claims of novel field induced Weyl states deep in the TI phase in Bi$_{1-x}$Sb$_x$.

## Supplementary Information

Supplementary Information.
