# Supplementary material for: Anomalous negative longitudinal magnetoresistance and violation of Ohm's law deep in the topological insulating regime in Bi1-xSbx
Source: Sci Rep. 2021 Apr 22;11:8756. doi: 10.1038/s41598-021-87780-0 (PMC8062501; doi:10.1038/s41598-021-87780-0)
Supplement: Supplementary file 1 — Supplementary Information. [file 41598_2021_87780_MOESM1_ESM.pdf]

Anomalous negative longitudnal magnetoresistance deep in the topological insulating regime in $Bi_{1-x}Sb_x$

Amit, R. K. Gopal, and Yogesh Singh

Department of Physical Sciences, Indian Institute of Science Education and Research (IISER) Mohali, Knowledge City, Sector 81, Mohali 140306, India.

Materials : The homogeneity of the $Bi_{1-x}Sb_x$ single crystals is very important. If for example, there is a mixture of phases with a small and large x, then the transport may be dominated by the small x phase since it has a smaller band-gap. We demonstrate the excellent homogeneity of our crystals by showing the energy dispersive x-ray spectroscopy (EDX or EDS) results on our x = 0.16 crystal. The EDS was measured on several individual spots on the crystal as well as on several areas of different sizes on the same crystal as shown in Fig.1(a-d).

Fig1
(a)

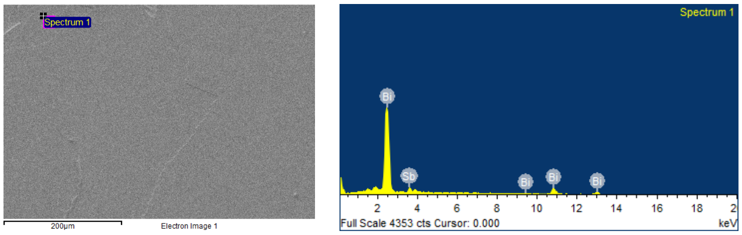

Element	Weight%	Atomic%
Sb L	9.54	15.32
Bi M	90.46	84.68
Totals	100.00	

(b)

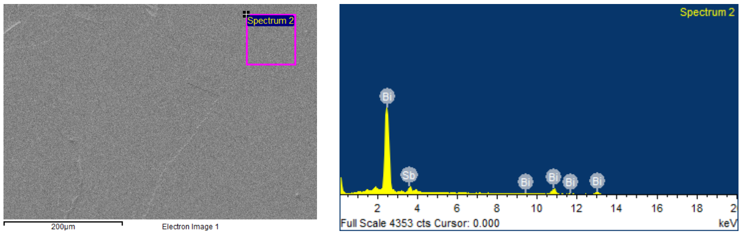

Element	Weight%	Atomic%
Sb L	10.81	17.22
Bi M	89.19	82.78
Totals	100.00	

(c)

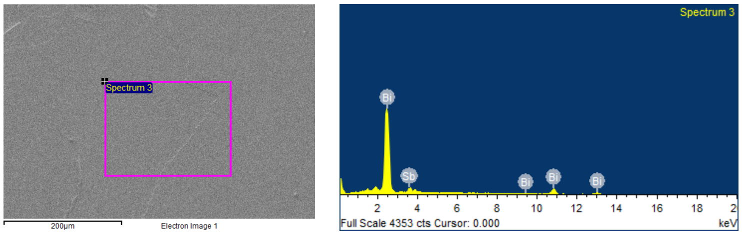

Element	Weight%	Atomic%
Sb L	10.38	16.59
Bi M	89.62	83.41
Totals	100.00	

(d)

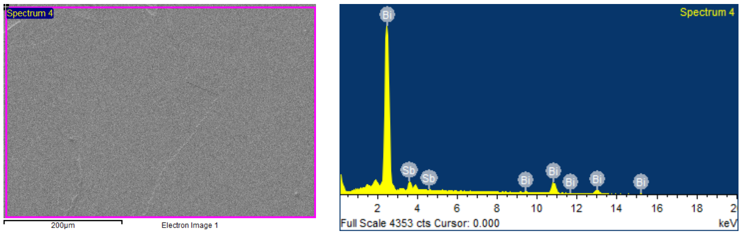

Element	Weight%	Atomic%
Sb L	9.64	15.47
Bi M	90.36	84.53
Totals	100.00	

The first column In Fig1.(a –d) shows the surface of the nominal $Bi_{0.84}Sb_{0.16}$ crystal and the pink square marks the area measured. The second column shows the spectrum that demonstrates the presence of both Bi and Sb in the crystal. The third column shows the estimated atomic percentage of each element. The result is that there is only about a 6% variation in the value of x i.e. the actual value with error is $x = 0.16 \pm 0.01$. Thus, we have demonstrated that the $x = 0.16$ crystal is homogenous in its composition.

Similar measurements were performed for each x and every composition was found to be homogenous to within ~5% of the target composition.
